# Field cancerization in dermatology

**DOI:** 10.1590/1806-9282.2024S113

**Published:** 2024-06-07

**Authors:** Renata Ferreira Magalhães, Thais Helena Buffo, Heitor de Sá Gonçalves, Carlos Baptista Barcaui, Aparecida Machado de Moraes

**Affiliations:** 1 Universidade Estadual de Campinas, Clinical Hospital, Division of Dermatology – São Paulo (SP), Brazil.; 2 Universidade Estadual de Campinas, Clinical Hospital, Department of Dermatology Surgery and Skin Cancer, Division of Dermatology – São Paulo (SP), Brazil.; 3 Center for Health Dermatology Dona Libânia – Fortaleza (CE), Brazil.; 4 Universidade Federal do Rio de Janeiro – Rio de Janeiro (RJ), Brazil.

## INTRODUCTION

The concept of the cancerization field was described in 1953 when a group of pathologists, studying malignant neoplasms of the mouth, detected atypical cells far from the main malignant lesion. It could be assumed that there was a carcinogenic stimulus, with modifications already existing in the nucleus of the cells, in apparently normal skin.

This change became evident where areas intensely exposed to the sun showed a frequent and progressive appearance of malignant and pre-malignant lesions^1,2^.

The definition of a field cancerization (CC) in dermatology is not yet fully established and is based on the visible identification of signs of sun damage associated with the finding of actinic keratoses and malignant epithelial tumors^3^.

The field cancerization by photo exposure is the most studied in dermatology.

The observation of this occurrence made it important to plan and approach patients with this alteration, as there was the potential for the condition to progress.

On the skin, in areas exposed to an aggressive factor such as sun exposure or radiation therapy, xerosis, atrophy, scaling, actinic melanosis, leukoderma, actinic keratosis, and tumors such as epidermoid carcinoma^1,4,5^.

In medical practice, highlighting this risk area is important for treatment, periodic and ongoing follow-up, and attention to the eventual appearance of more serious injuries.

**Figure 1:**
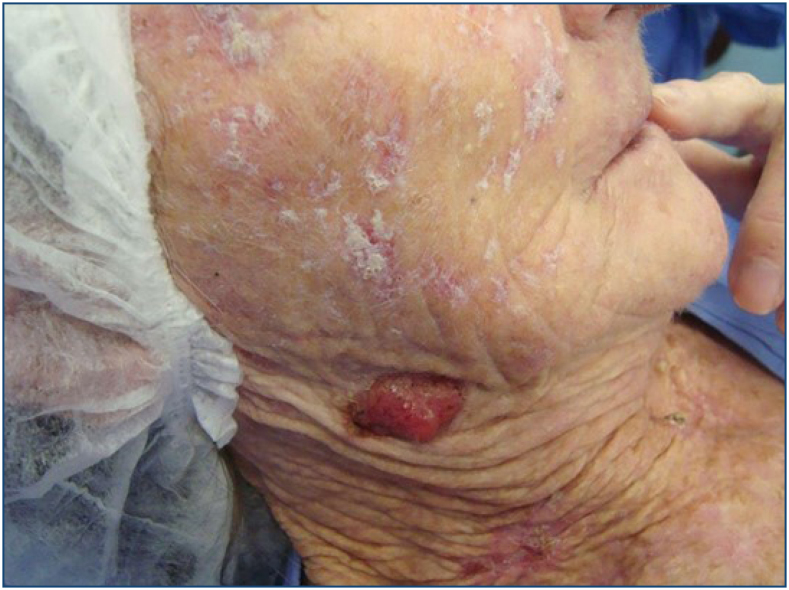
Multiple actinic keratoses on the face and squamous cell carcinoma on the neck.

There is still disagreement in the literature as to the exact field of cancerization, but some findings are considered highlights, such as actinic keratosis^1,4^ (Figure 1).

## ASSOCIATED FACTORS

**Figure 2:**
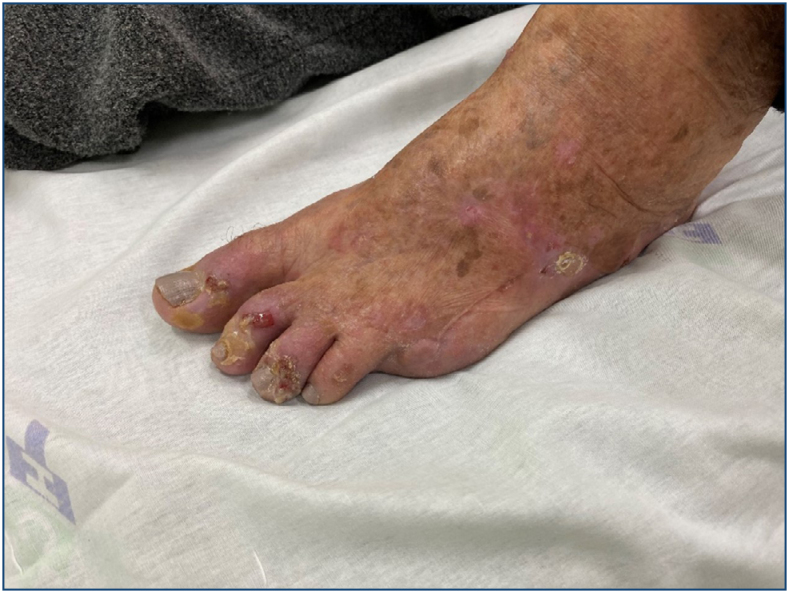
Foot intensely exposed to the sun, actinic keratoses, and carcinomas. Loss of finger due to a tumor.

Several factors may be associated with the development of a field cancerization, the most notable being chronic sun exposure. Sun exposure from childhood is considered and, even if the patient does not give correct information about the intensity and time of exposure, indirect data such as sports practices, free leisure activities, and rural work should be considered (Figure 2).

There is a greater risk in fair-skinned individuals, older patients, and immunosuppressed patients such as transplant patients.

A history of a lesion treated with a diagnosis of squamous cell carcinoma puts the region at greater risk of developing others^4,5^.

## CLINICAL MANIFESTATIONS

The lesion that stands out on examination is actinic keratosis. It can be a precursor to squamous cell carcinoma or show histopathological atypia in the process of developing into carcinoma.

Actinic keratosis can be single or multiple, as papular lesions with hyperkeratosis and erythema.

They may be painful, have a burning sensation, or feel like a "thorn in the skin." They may be more palpable than visible, giving a feeling of local roughness.

Hyperkeratosis, which can be pronounced, suggests the shape of a cutaneous horn.

There are agglomerated, confluent actinic keratoses, forming extensive hyperkeratotic plaques^5-7^.

The presence of actinic keratosis in a region of the skin requires careful observation as other manifestations suggestive of a field cancerization can be found such as xerosis, desquamation, actinic melanosis, and tumoral lesions of epidermoid carcinoma.

This can present as a nodule, tumor, vegetating lesion, and infiltration in varying sizes.

The areas most affected are those exposed to the sun, such as the bald head, face, upper limbs, and neckline.

There are attempts in the literature to grade actinic keratoses and to establish a relationship between higher keratosis rates and a higher risk of developing epidermoid carcinoma^1,8^.

## HISTOPATHOLOGICAL ASPECTS

**Figure 3:**
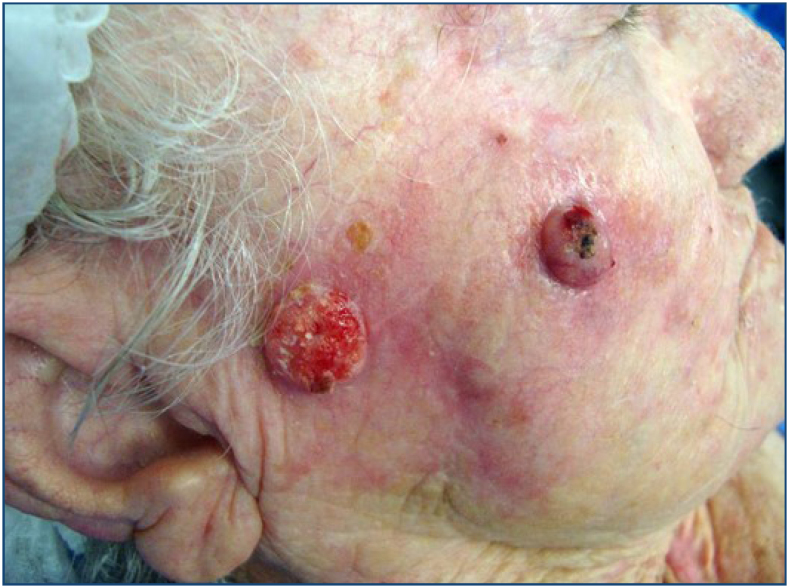
On the face, squamous cell carcinomas at different stages of development.

The histopathological study of the field cancerization refers to the clinical lesions studied, such as actinic keratoses or carcinomas (Figure 3).

It is possible to observe epidermal changes of atypia and architectural irregularities along the path of clinically normal areas in a large field.

**Figures 4 and 5:**
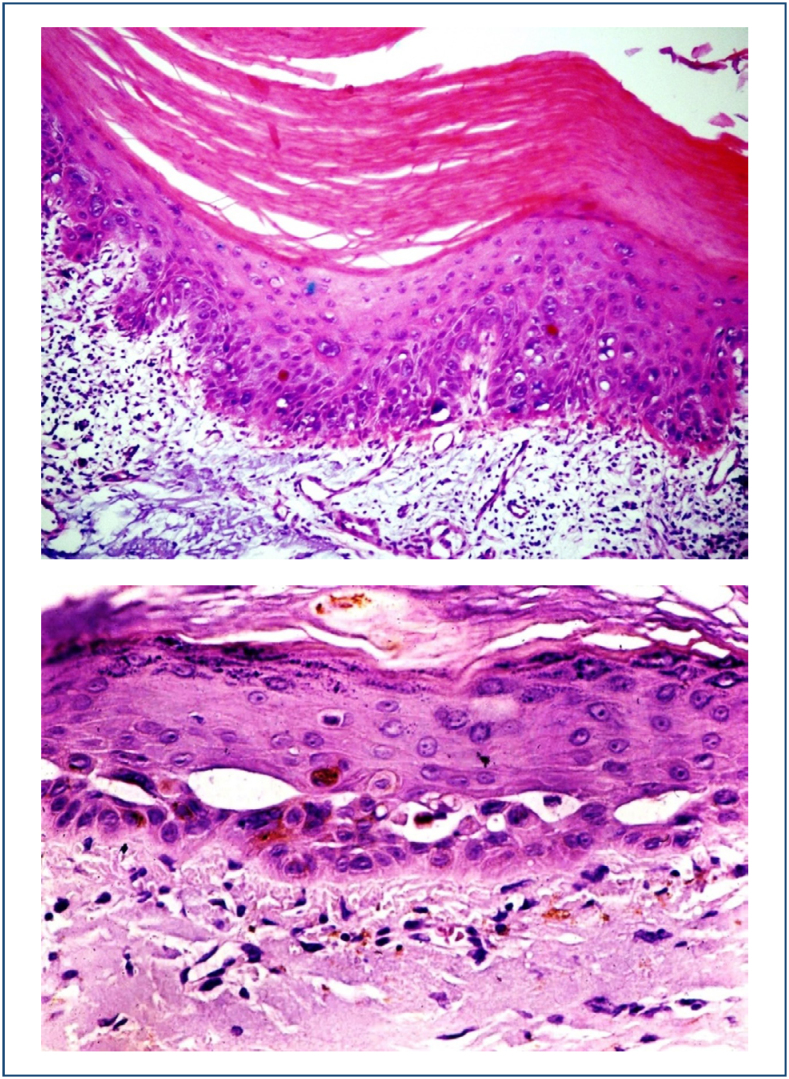
Histopathological view of actinic keratosis showing epidermal nuclear atypia, hyperkeratosis, and structural disarray.

Histopathological view of actinic keratosis showing epidermal nuclear atypia, hyperkeratosis, and structural disarray (Figures 4 and 5).

## TREATMENTS

Treatments for the field cancerization have evolved a lot in recent years, given the demand for the number and diversity of new cases.

Treatments can be single or combined, starting with one approach and extending to others depending on the presentation, number of lesions, and severity.

The following steps are suggested in the treatment of the field cancerization:

- Define the area of involvement and the number of lesions;
- Collect samples from lesions with the clinical appearance of carcinomas, hypertrophic actinic keratoses, or lesions clustered in plaques;
- If epidermoid carcinoma is confirmed, prioritize its treatment, preferably surgical;
- Establish an ablative treatment program for multiple, hypertrophic actinic keratosis lesions;
- Propose treatment of the field with non-ablative methods;
- Establish regular treatments, at least annually;
- Propose preventive treatments (Table 1).

**Table 1:** Principal treatments^4,8-10^.

Ablative treatments	Topical treatments
Conventional surgery and MMS	5-Fluorouracil
Curettage and electrocoagulation	Imiquimod
Cryosurgery	Diclofenac sodium
Laser	Retinoic acid
Photodynamic therapy	Ingenol mebutate
Dermabrasion	

### Ablative treatments

Ablative treatments include conventional surgery, cryosurgery, and laser therapy.

Conventional surgery is indicated for the excision of clinically and histologically diagnosed carcinomas. It is recommended to excise all carcinoma lesions with a safety margin of at least 0.4 cm beyond the safety margin^11,12^.

It is well known that the safest method for excising these tumors is Mohs micrographic surgery, which is performed with microscopic control of the margins. The cure rate with this method is known to be higher than with conventional surgery.

According to clinical and histopathological criteria, epidermoid carcinoma is currently classified into low and high risk^11-13^. Both can be found in a field cancerization and will be treated according to risk. In the case of established carcinoma lesions, these are considered a priority for treatment.

For low-risk carcinomas and actinic keratoses, cryosurgery with liquid nitrogen is recommended.

**Figure 6:**
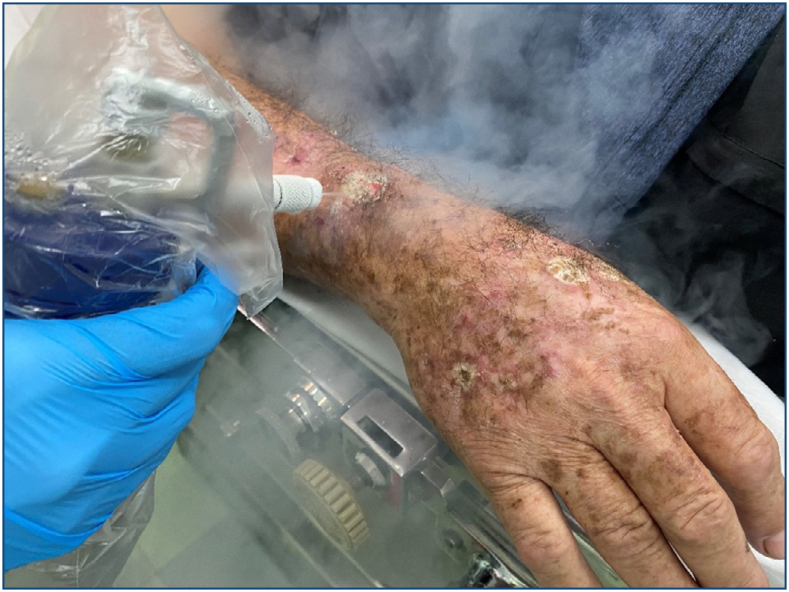
Cryosurgery with liquid nitrogen in actinic keratoses of the field cancerization.

Cryosurgery is performed by applying a jet of liquid nitrogen to the lesion, freezing it, and resulting in coagulation necrosis. The lesion will be eliminated once the necrosis and crust have been removed^8,14^ (Figure 6).

**Figure 7:**
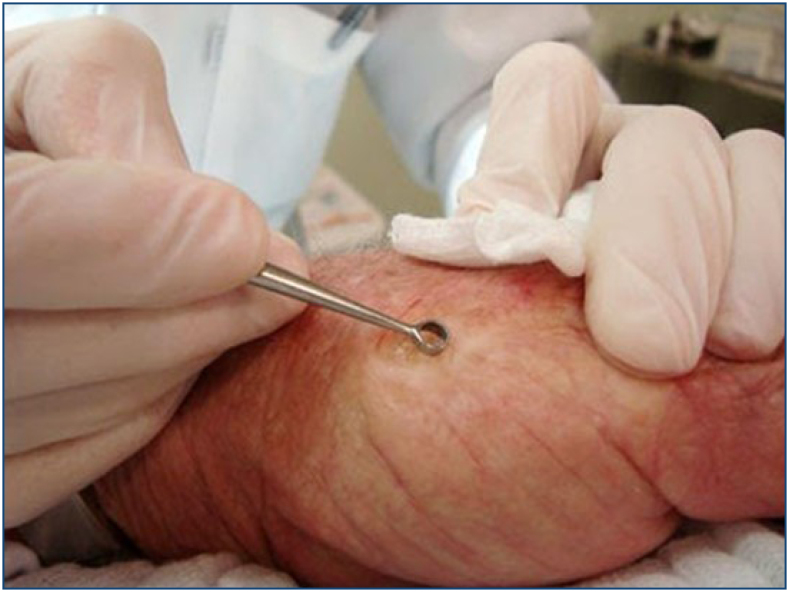
Actinic keratoses curettage.

Curettage and electrocoagulation are also described. This method involves curettage of the lesion, which is more suitable for actinic keratoses, ulcer formation, and subsequent electrocoagulation of the wound. In addition to hemostasis, the electric current promotes coagulation necrosis, which helps to eliminate the atypical cells located there^3,5^ (Figure 7).

In recent years, the use of photodynamic therapy for cancerizable areas has been well described.

This involves applying a photosensitive substance, for example, methyl 8-aminolevulinate, to the affected area. The affected area should be prepared by light curettage of the most prominent keratoses and the photosensitive material is incubated under occlusion for 1 h. Afterward, a red light source can be applied or the patient is instructed to take mild sun exposure (daylight method). The affected area will be sensitized by the reaction of the drug with the light and the lesions that absorb the product will react more intensely at the most impregnated points. The result will be an erythematous area, followed by peeling and the elimination of actinic keratoses.

This method has been advocated for field cancerization areas and superficial epithelial tumors, squamous cell, and basal cell carcinomas. It is recognized that possible clinically unnoticeable lesions will be eliminated with this method^3,5,15^.

There are references to the use of ablative laser therapy, such as CO_2_ laser and dermabrasion, in the treatment of keratoses and fields cancerization. These resources are considered when treating heavily affected areas and hypertrophic lesions.

The knowledge and availability of different therapeutic methods increase the resolutions in different presentations of the disease^4^.

### Topical treatments

There are various topical treatments available, recommended mainly for superficial actinic keratosis lesions and basal cell and squamous cell carcinomas. For the drugs described below, prior curettage of the hypertrophic lesions is recommended to improve penetration of the active products.

The following therapies are mainly described.

#### 5-Fluorouracil

An antimetabolite that inhibits DNA synthesis, presented in 0.5–5% cream, promotes deposition in epithelial cells in accelerated turnover and their consequent apoptosis. The area of application becomes erythematous and edematous with visible scaling in the most active lesions. Daily applications are recommended for 4 weeks^3,8,16^.

#### Imiquimod

**Figure 8:**
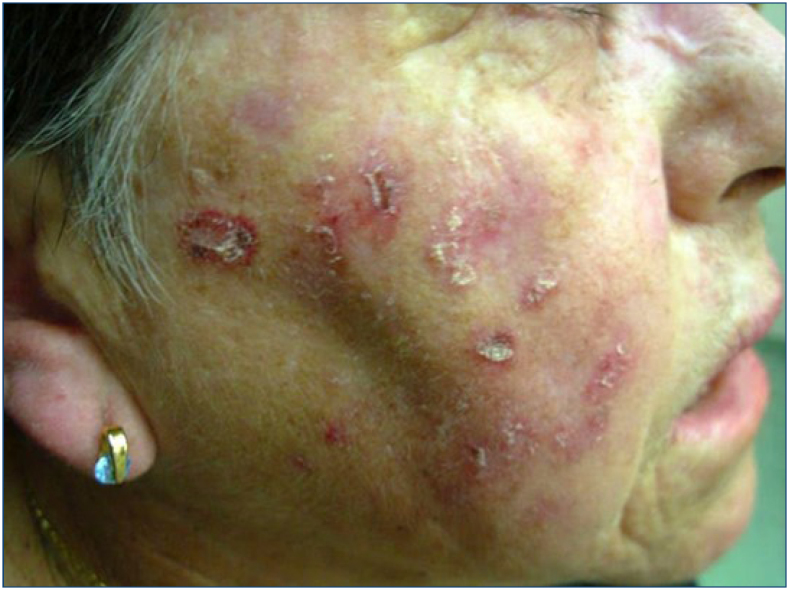
Patient using imiquimod on the seventh day.

Imiquimod is a 3.75–5% cream, non-specific immunomodulatory agent. Applications are recommended three times a week for 6–16 weeks (Figure 8).

Both of the above drugs promote an intense inflammatory reaction with erythema, edema, exudation, and crust formation. The continuity of applications depends on the patient's tolerance^8^.

#### Diclofenac sodium

It is presented in 3% aqueous gel formulations associated with 3% hyaluronic acid; it inhibits prostaglandin synthesis, elevated in lesions due to actinic damage; and its use should be prolonged and recommended twice a day for 3 months. Improvement of actinic keratosis lesions is slow and is estimated to be relevant in preventing new ones^6,7,17^.

#### Ingenol mebutate

It is prepared as a gel at 0.05 and 0.1% and applied to the face or body for 2–3 days. It induces necrosis of abnormal keratinocytes, an inflammatory reaction, and stimulates the production of anti-tumor antibodies. The advantage of this treatment is its short course, which does not lead to the patient discontinuing it. It also produces an intense inflammatory reaction and its correlative signs on the skin^8-10^.

### Systemic treatments

Although it is not the scope of this text, it should be emphasized that when squamous cell carcinomas are found that are clearly developed or with the possibility of distant metastases, they should be evaluated for systemic therapy.

Treatment includes chemotherapy and immunotherapy drugs such as cemiplimab^12^.

## PREVENTION

The constant search for preventive treatments for multiple actinic lesions has been intense worldwide, considering the limitations and costs that this condition produces in populations, especially those with fair skin and those who frequent environments with high sun exposure^17,18^.

The prevention of cancerizable skin lies mainly in protection from solar ultraviolet radiation. Early protection, from childhood onwards, is recognized as a major factor against the development of the field^18,19^.

The use of appropriate clothing and sunscreen in its different presentations and sports and work habits during periods of lower radiation are recognized as important to avoid the development of the field cancerization and its skin lesions.

Current studies have indicated nicotinamide in the chemoprevention of skin cancer. It is the amide form of vitamin B3. It has been implicated in maintaining genomic stability and may have beneficial effects on skin aging and tumor development^20-22^.

The authors described a reduction in the appearance of malignant epithelial tumors in patients who used nicotinamide 500 mg twice a day compared with placebo^5,20,21^.

Periodic follow-up of patients who have treated a field cancerization is necessary, at least annually and, if new lesions appear, this period should be brought forward.

It is recognized that actinic keratoses are recurrent.

Periodic courses of topical treatments are indicated for the chemoprevention of squamous cell carcinoma, as described by Weinstock et al.^16^.

Recent research has pointed to the maintenance of the cutaneous microbiome in the chemoprevention of cutaneous carcinomas and suggests studies to be carried out on the involvement of diets, vitamin D, and microbial therapies^23^.
